# Cracks observed to propagate discontinuously on the millisecond timescale

**DOI:** 10.1107/S2052252516002359

**Published:** 2016-02-25

**Authors:** Brian K. Tanner

**Affiliations:** a Department of Physics, Durham University, South Road, Durham, DH1 3LE, United Kingdom

**Keywords:** silicon wafers, crack propagation, X-ray diffraction imaging, phase-contrast X-ray imaging, time-resolved studies

## Abstract

Ultra-fast diffraction and phase contrast imaging experiments on crack propagation in silicon, reported in the current issue of **IUCrJ**, are reviewed in the light of our present knowledge and its industrial importance.

The simple, controllable, cleavage of (001) oriented silicon wafers into rectangular die by scribing in the <110> directions hides some complex and imperfectly understood fracture mechanics. There can be substantial economic consequences. For example, catastrophic fracture during high temperature processing of silicon can take place *via* complex crack paths (Tanner *et al.*, 2015 ▸; see also Fig. 1 ▸) that result in small wafer fragments, which are difficult to remove, contaminating the processing tool. The time associated with such a production line halt is a multi-million dollar cost within the semiconductor industry. We have shown that the crack paths macroscopically follow the stress contours in the wafer associated with the thermal gradients in the processing tool (Tanner *et al.*, 2012 ▸). However, the prediction of the crack paths at a microscopic level is more challenging.

At the very simplest level, one can calculate the quasi-static cleavage energy of specific lattice planes using full-density functional molecular dynamic simulations (Pérez & Gumbsch, 2000 ▸). It is found that the {111} planes have the lowest energy at 2.88 J m^−2^, while the {110} planes have an energy of 3.46 J m^−2^. However, in a standard silicon wafer, the {110} planes are perpendicular to the (001) surface whereas the {111} planes are inclined at an angle of 35.26°. The result is that the larger surface area of the {111} planes increases the cleavage energy by a factor of 1.225, making fracture on the {110} planes favourable, as is observed during the fabrication of semiconductor device die and microelectromechanical sensor (MEMS) devices.

This {110} cleavage occurs for low crack velocities (below about 1500 m s^−1^), but when the crack velocity becomes high, (above about 3000 m s^−1^), propagation entirely on the {111} planes is observed (Sherman, 2006 ▸). At intermediate velocities, propagation starts on the {110} planes but then switches to {111}, driven, it is suggested, by the energetics of phonon emission. Hybrid classical/quantum mechanical molecular dynamics simulations do indeed predict such behaviour (Kermode *et al.*, 2008 ▸).

An associated crack propagation phenomenon which is presently imperfectly understood, despite some elegant recent modelling work (Kermode *et al.*, 2015 ▸), is lattice trapping whereby cracks do not move uniformly. In a remarkable paper in this issue of **IUCrJ**, Alexander Rack, Mario Scheel and Andreas Danilewsky (Rack *et al.*, 2016 ▸) report novel X-ray experiments which may begin to help us understand this effect. They report ultra-high speed imaging experiments at ID19 of the European Synchrotron Radiation Facility in Grenoble in which they image a moving crack simultaneously by phase contrast and diffraction contrast. Creating a stress gradient by cooling part of the hot silicon wafer with a water jet, they have driven a crack, associated with indentation damage, over a distance of about 1 mm with an exposure time of 1.28 µs. The ultra-fast X-ray diffraction imaging (topography) reveals the development of the strain field as the crack propagates while the phase contrast image reveals the physical position of the crack at the same time. The mean crack velocity is low, typically 0.0028–0.055 m s^−1^, many orders of magnitude below that described by Sherman (2006 ▸). However, the images show that the crack moves in a series of jumps in which the crack moves at a speed greater than the 6 m s^−1^ resolution limit, followed by a stop time of between 1 and 2 ms. The motion is consistent between phase and diffraction contrast images.

Although the crack appears to propagate macroscopically in the 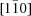 direction in the plane of the (001) wafer, the microscopic surfaces do not show smooth {110} cleavage. High-resolution *ex-situ* diffraction images show lines in the cleavage plane which correlate with the jumps in crack position revealed by the *in-situ* experiments. These steps correspond to micro-cleavage steps, mainly on inclined {111} and {110} planes.

It is quite extraordinary that over the 57 years from the invention of X-ray topography by the late Andrew Lang, exposure times have gone from the best part of a day to a microsecond. The key, of course, has been improvements in both sources and detectors. Rack, Scheel and Danilewsky point out that a camera with a frame transfer rate of 10 million images per second has already been successfully tested at the ID19 beamline at the ESRF and that, with the diffraction-limited synchrotron light sources presently under construction, an increase in brilliance of two orders of magnitude can be expected in the next few years. They suggest that these combined developments may enable us genuinely to study the dynamics around a propagating crack in the near future.

## Figures and Tables

**Figure 1:**
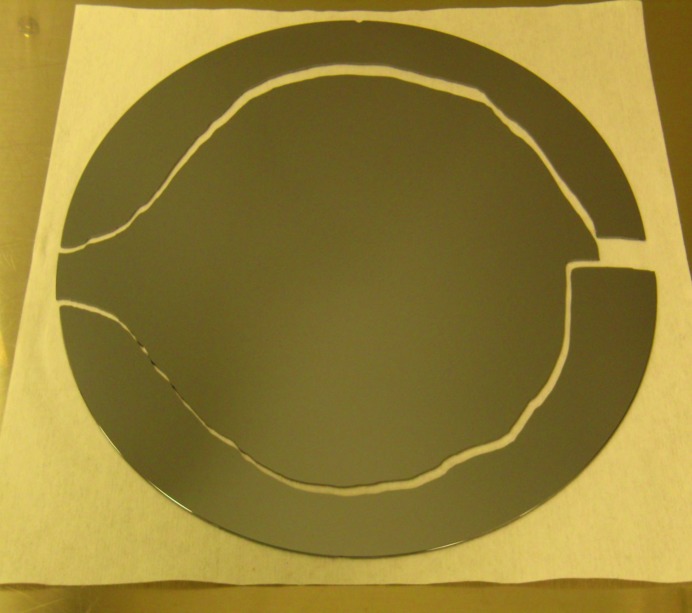
Complex fracture of a (001) silicon wafer following rapid thermal annealing (courtesy J. Garagorri, PhD thesis, Universidad de Navarra, San Sebastian, 2014).
